# Anti-Infectious Plants of the Thai Karen: A Meta-Analysis

**DOI:** 10.3390/antibiotics9060298

**Published:** 2020-06-02

**Authors:** Methee Phumthum, Henrik Balslev

**Affiliations:** 1 Department of Pharmaceutical Botany, Faculty of Pharmacy, Mahidol University, Bangkok 10400, Thailand; 2 Sireeruckhacharti Nature Learning Park, Mahidol University, Nakhon Pathom 73170, Thailand; 3 Department of Biology, Faculty of Natural Science, Aarhus University, 8000 Aarhus, Denmark; henrik.balslev@bios.au.dk

**Keywords:** ethnobotany, ethnomedicinal plants, infectious diseases, antimicrobial, bacterial, fungal, viral, *Tinospora crispa*, *Cleidion javanicum*, *Litsea cubeba*

## Abstract

Pharmacology has developed many drugs to treat infections, but many people, especially in developing countries, cannot afford to purchase them, and still depend on traditional knowledge and local medicinal plants to fight off infections. In addition, numerous microbes have developed resistance to the pharmaceutical drugs developed to fight them, and for many, such as Covid-19, effective drugs remain to be found. Ethnomedicinal knowledge is useful, not only for local people as a source of medicine for primary health care, but also for new pharmacological discoveries. This study aimed to identify the plants that the Karen, the largest hill-tribe ethnic minority in northern and western Thailand, use for treatments of infectious diseases. We present a meta-analysis of data from 16 ethnobotanical studies of 25 Karen villages with the aim of understanding traditional knowledge and treatments and point to potential plants for further pharmacological development. The Karen used 127 plant species from 59 plant families to treat infections and infectious diseases. The Cultural Important Index (CI) showed that the Leguminosae, Euphorbiaceae, Asteraceae, Lauraceae, Apocynaceae, Menispermaceae, and Lamiaceae were the most commonly used families. As for species, *Cleidion javanicum*, *Tinospora crispa*, *Litsea cubeba*, *Aesculus assamica*, *Tadehagi triquetrum*, *Senna alata*, *Tithonia diversifolia*, *Embelia sessiliflora*, and *Combretum indicum* were the most commonly used in treatments of infectious diseases. We suggest that these plant species should be the first to be pharmacologically tested for possible development of medicines, and the remaining species registered should subsequently undergo testing.

## 1. Introduction

Infectious diseases have troubled humans through history and killed many all over the world, especially when the diseases were new- and re-emerging. In the 16th century, smallpox and Cocoliztli devasted large numbers of people in Mexico [1]. Ever since, influenza has caused huge numbers of deaths in many countries [2]. Over the last decade we have seen outbreaks of deadly infectious diseases such as SARS, H5N1, H1N1, MERS, Ebola, and Zika. Currently, while we are preparing this paper, Covid-19, a new emerging disease, has become a pandemic and killed thousands of people globally. However, deadly diseases are not only viral infections. Bacterial infectious diseases, such as foodborne *Escherichia coli* O104: H4 and nontyphoidal *Salmonella*, are also severe and sometimes fatal [3].

From an evolutionary perspective, we now face serious health problems caused by antibiotic-resistant strains of for example *Staphylococcus aureus* [3]. It is expected that in this century infectious diseases will cause more deaths than it did in the past because of the rapid growth of the world population, which is predicted to reach eight billion, of which more than half will live in crowded areas [3]. Nowadays, cheaper air transportation allows more than 100,000 people to fly on regional and global routes each day, which contributes to spreading diseases. Microorganisms move rapidly under warm conditions. By 2050, it is expected that approximately 50% of the world’s population, including 60% of the world’s children, will reside in the tropics [4]. Most tropical regions have poor developing countries with inadequate medical and public health care systems. Consequently, the mortality due to infections will be very high in those areas. Infectious diseases also make a considerable impact on the economy and the environment. The Avian Influenza or bird flu, caused by the virus H5N1 in 2004, caused the death of 12 percent of all domestic birds in Vietnam; many of them were deliberately killed to prevent the disease from spreading. Globally, approximately 0.2% of laborers in the poultry sector lost their jobs. In 2006, it was estimated that the global economic impact of this influenza was over $1.5 trillion corresponding to more than 3% of the world’s GDP. Therefore, in the near future, alternative and more powerful medicines will be needed to cure existing and new infectious diseases.

Many modern pharmaceuticals were derived from plant sources and metabolic scaffolds, and a significant number were developed based on ethnobotanical knowledge [5]. In classical discoveries, drug researchers found hundreds of medicines, including opium, quinine, aspirin, and digitoxin. *Opium* was known as a medicine for millennia. The world’s oldest clay tablet was made using this plant by the Sumerians and it was used for pain relief [6]. Today drugs such as morphine, codeine, and other opiate derivatives are extracted from opium as analgesic agents [7]. *Quinine* is another example of a high impact drug, which was until recently the most potent medicine used to treat malaria [8]. Quinine was listed as the gold standard by the WHO in malaria treatment [9]. However, after the discovery of *artemisinin*, the WHO is no longer recommending quinine as a first-line to treat malaria, and it should be used only when artemisinin is unavailable, due to quinine’s undesirable side effects [10]. Artemisinin was also developed from botanical traditional use and discovered by the investigation of more than 2000 herb preparations from the Chinese pharmacopeia. An extract from *Artemisia annua* L. emerged as an outstanding inhibitor of parasitic growth, and the enriched extract turned out to be non-toxic. The active ingredient, artemisinin, was identified [11] and is now used globally to cure malaria. After becoming the first-line of essential medicines recommended by the WHO [9,10], the discoverer, Professor Youyou Tu, was awarded the noble prize in 2015. In 2001, Fabricant and Farnsworth listed hundreds of drugs developed from traditional knowledge [5]. These examples of medicinal discoveries demonstrate the value of traditional medicinal knowledge.

Thailand is botanically very rich [12,13] and at the same time a culturally diverse country with more than 70 ethnic minorities in addition to the Thai speaking majority [14]. There are many studies of medicinal plants from many of these ethnic minorities [15], but only a few of them focus on infectious diseases [16]. The Karen is an ethnic minority group—the largest of the hill-tribes in Thailand [14]. They migrated from Myanmar because of wars in the 1800s [17] and now live in 16 provinces in northern and western Thailand [18]. Thai Karen are farmers and their villages are located close to forests and streams [19], where most of them practice their original animism even if some have converted to Buddhism and Christianity [20]. In their remote settlements, plants are a primary source of medicines for their health care and the Karen possess tremendous amounts of traditional ethnomedicinal plant knowledge [21,22]. The ethnomedicinal knowledge of Karen is the most extensively studied among ethnic minorities in Thailand [23]. The ethnobotany of Thai Karen was first published in 1993 [24] and since then several studies have been conducted in many Karen villages, especially in Chiang Mai province. However, none of these studies were focused on medicinal plants used to treat infectious diseases. Using the Cultural Importance index (CI), which measures how commonly a particular use is mentioned among villagers, and doing a meta-analysis of 25 villages of the Karen traditional knowledge, we aim to find medicinal plants used for treatments of infection diseases. Specifically, we ask: (1) which plant species and families were most commonly used by the Karen in Thailand for treatments of infectious diseases? and (2) which plant species have the potential to be developed into a new medicine?

## 2. Results

**Figure 1:**
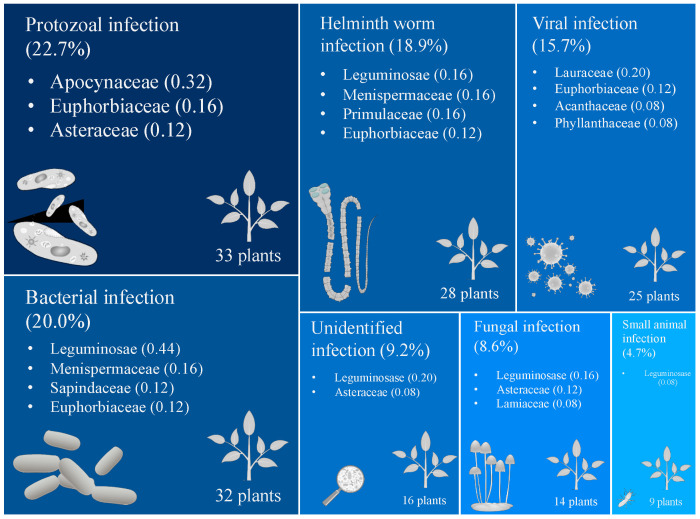
Percent of use reports mentioned for each infection type treated with medicinal plants by the Karen in Thailand. Darker blue color and larger infection type area indicate higher number of use reports. Plant families mentioned under an infection type area are those with high CI values and the number in the parentheses after the family names indicates the CI value of the family. The numbers of plants in the lower right corners of the areas represent total numbers of plant species used for treatments of the infection types.

In the 16 references, that covered 25 Karen villages, we found 185 reports of plants, which included 127 species in 59 plant families, used to treat infectious diseases. Most reports mentioned uses for treatments of protozoal (23%), bacterial (20%), helminth worm (19%), and viral (16%) infections. There were also 16 reports of uses for fungal infection, and nine reports for small animal infestation (Figure 1 and Supplementary materials 2 Table S1). Seven plant families had high total CI values (Leguminosae, Euphorbiaceae, Asteraceae, Lauraceae, Apocynaceae, Menispermaceae, Lamiaceae), but only few of them had high CI values for each infection type. Apocynaceae was the most used family for the treatment of protozoal infections. Leguminosae was the top ranked family for treatments of bacterial, helminth worms, and fungal infections, and it was also important for treating small animal infestations. Lauraceae had the highest CI value for viral infection. Other families, e.g., Euphorbiaceae, Asteraceae, and Menispermaceae, had high CI values for treatments of various infection types (Figure 1 and Supplementary Materials 2 Table S1).

The Karen used 33, 32, 28, 25, 16, 14, and 9 plant species, respectively, for treatments of protozoal, bacterial, helminth worm, viral, unidentified, fungal infections and small animal infestations (Figure 1 and Supplementary Materials Table S1). *Cleidion javanicum* and *Tinospora crispa* had the highest total CI values. *Cleidion javanicum* was used to treat bacterial, protozoal, and viral infections, whereas *T. crispa* was used to treat bacterial, helminth worm, protozoal, and unidentified infection types. *Litsea cubeba* was another plant with high CI value for treatments of infectious diseases. The Karen used this species to treat bacterial, fungal, protozoal, and viral infections. Three species were used to treat four infection types, namely *Litsea cubeba* (bacterial, fungal, protozoal, viral), *Tinospora crispa* (bacterial, helminth worm, protozoal, unidentified), *Rotheca serrata* (fungal, helminth worm, protozoal, unidentified). Six species were used to treat three infection types—*Cleidion javanicum* (bacterial, protozoal, viral), *Aristolochia tagala* (helminth worm, unidentified, viral), *Acacia caesia* (bacterial, helminth worm, viral), *Cajanus cajan* (bacterial, small animal, unidentified), *Acacia concinna* (bacterial, fungal, unidentified), and *Scoparia dulcis* (bacterial, fungal, protozoal). Nine species were used to treat two infection types, and the remaining species were used to treat a single infection type (Table 1).

Different plant species were used to treat different infection types. For bacterial infection, plants that had the top CI values were *Tinospora crispa*, *Aesculus assamica, Cleidion javanicum*, and *Tadehagi triquetrum*. *Senna alata* and *Tithonia diversifolia* were recognized as powerful plants for treatment of fungal infections. Four plants—*Embelia sessiliflora*, *Tinospora crispa*, *Tadehagi triquetrum*, and *Combretum indicum*—had high CI values for treatment of helminth worm infection. *Cleidion javanicum* and *Rauvolfia verticillata* could be potent for treating protozoal infection. *Archidendron jiringa* had the highest CI value for treating unidentified infectious diseases. Lastly, three species—*Litsea cubeba*, *Cleidion javanicum*, and *Flueggea leucopyrus*—were the top species used for treatments of viral infection (Table 1).

## 3. Discussion

Our database for the treatment of infectious diseases is the largest for Thailand, and possibly in general, in terms of the number of studied villages. Excluding the number of reports for fever treatments, our study covers more plant species used for treating infectious diseases in Thailand than previous studies. We have shown that 59 plant families were found to have species that were used in treatment of infectious diseases, but only a few of them covered the overwhelming majority of uses. This was especially true for Leguminosae, Euphorbiaceae, Asteraceae, Lauraceae, Apocynaceae, Menispermaceae, and Lamiaceae, some of which were used to treat multiple kinds of infectious diseases. These same plant families are known for their many medicinal uses in Thailand [15,16,17,18,19,20,21,24,25] and elsewhere [26,27,28,29], and they are particularly known for their antimicrobial properties [30,31,32,33,34,35].

When compared by their cultural importance, as expressed in the CI index, *Cleidion javanicum*, *Tinospora crispa*, and *Litsea cubeba* were the most commonly used for treatments related to infectious diseases in various categories. In a previous study of Thai medicinal plants in general, *Tinospora crispa*, *Cleidion javanicum,* and *Litsea cubeba* were also ranked among the top species among 2187 with the highest Species Use Value (UV) [15].

Fever is also a sickness caused by infections, especially viral infections. However, the comparison between ethnomedicinal plants used in this study and the ones used to treat fever by the Karen [22] shows interesting information. Two hundred and thirteen plant species were listed in both studies—127 plants were used to treat general infections and 125 species used for fever. However, only 39 plant species were used in both studies (Supplementary Materials Table S2). Although fever, in general, is caused by infections, the human body creates sophisticated mechanisms to recover. Hence, medicinal plants that the Karen used to treat fever were not only used for antimicrobial infections but also for enhancing the recovery mechanism. Some species might be used as immunostimulants, for example, *Andrographis paniculata* [36], while other species might enhance mechanisms related to body temperature. However, there were a few species that had high CI values reported by both studies—*Tadehagi triquetrum, Scoparia dulcis, Rotheca serrata, Melicope glomerata, Cleidion javanicum, Chromolaena odorata*, and *Alstonia scholaris* (Table 1 and [22]). Therefore, these species might have efficacy for anti-infectious diseases.

*Tinospora crispa,* which had the top CI value in this study, is also used for treatments of infectious diseases in other parts of the world, and its extracts have a broad range of pharmacological activities [37]. For antimicrobial activities, *T. crispa* includes effects on *Staphylococcus aureus, Streptococcus pneumonia, Bacillus cereus, Escherichia coli, Pseudomonas aeruginosa, Candida albicans,* and *Saccharomyces cerevisiae* [38,39]. The plant produces a variety of secondary metabolites including lactones, steroids, flavonoids, lignan and alkaloids. The compound names and structures are shown in [37]. Specifically, *T. crispa* produces furanoditerpenes, which are characteristic of the species [37]. *Cleidion javanicum* and *Litsea cubeba* also have the potential for antimicrobial activities. *Cleidion javanicum* produces various essential oils that have antimicrobial activities. The major compounds are ethyl linoleolate, hexadecanoic acid, and trans-phytol [40]. Essential oils from *L. cubeba*, citral and limonene, have antibacterial and antifungal activities [41].

This study lists 127 Karen medicinal species used for treatments of infectious diseases, but only a few of them were used for many different types of infection and several were used only for a single infection type (Table 1). Although these species had low CI values, other studies have shown that some of them have antimicrobial activities, such as *Acmella oleracea* [42], *Euphorbia heterophylla* [43], *Peperomia pellucida* [44], and some other species listed in Table 1.

This study shows another interesting result related to helminth infection and protozoal infection. According to the Karen lifestyles and limitations to access local health centers, it was expected that the Karen would have several plants with high CI values for treatments related to these infectious types. Only *Embelia sessiliflora* had a high CI value. However, in total, the Karen had 33 species (26% of all useful species) used for protozoal infections and 28 species (22%) for helminth worm infection (Table 1). These types of infections are common but not severe, for example, intestinal worm infection. It is possible that many plants, but non-specific species, have potential as treatments, which leads to the use of a variety of plants for treatments. In addition, the study found that each village in Thailand has unique ethnomedicinal knowledge [45]. Having low CI values but a wide variety of plants used for treatments related to these two infectious types would be because the informants developed their knowledge for the treatments.

This meta-analysis identified a large number of medicinal plants that were used for treatments of sicknesses related to infections and infestations among the Karen ethnical minority in Thailand. Although the Karen population make up less than two percent of the Thai population [18], the people have considerable plant ethnomedicinal knowledge [21]. We showed that a limited number of plant families (Leguminosae, Euphorbiaceae, Asteraceae, Lauraceae, Apocynaceae, Menispermaceae, and Lamiaceae), (Figure 1 and Supplementary Materials Table S1) are the most important anti-infectious plants. The species with the highest potential for the development of new medicines for treatments of infectious diseases were *Cleidion javanicum*, *Tinospora crispa*, *Litsea cubeba*, *Aesculus assamica*, *Cleidion javanicum*, *Tadehagi triquetrum*, *Senna alata*, *Tithonia diversifolia*, *Embelia sessiliflora*, *Tinospora crispa*, *Tadehagi triquetrum*, and *Combretum indicum* (Table 1).

Although our study included the largest dataset for medicinal plants used by Karen for treatments of infectious diseases in Thailand, our data most likely does not represent all plants used by Karen for the treatments. Many used plants had very low CI values. This is a weakness of meta-analytical studies that depend on available data. Our dataset is limited as it covers only 25 of the approximately 2000 Karen villages spread over northern and western Thailand [18]. That number is only 1.5% of all Karen villages in the country. Another possible disadvantage is that ethnobotanical knowledge may be idiosyncratic and different from village to village as shown in a previous study [45]. Furthermore, a study found that that entire dataset on Thai ethnomedicinal plant species has not been completed [15]. This uncertainty can only be resolved by additional field surveys in other unstudied Karen villages.

## 4. Materials and Methods

Data were collected from published data in Thai university libraries, Thai online journals, online databases (Google scholar, Scopus, PubMed), and the database of the Thai Library Integrated System (www.tdc.thailis.or.th), which includes almost all post graduate student theses, scientific articles, and scientific reports from all higher educational institutions in Thailand. Repeated data were removed by screening data from journal articles that repeated student’s theses. Reports that did not use ethnobotanical methods for their studies, and reports that did not provide scientific plant names were also excluded. All scientific plant names were updated following The Plant List website (www.theplantlist.org), which is now merged into the World Flora Online website (http://www.worldfloraonline.org/). All plant uses for treatments of the category infections/infestations in the *Economic Botany Data Collection Standard* (EBDCS) [46] were included in this study. However, we excluded ethnomedicinal plant uses for treatment of fever because the symptoms are so complicated, i.e., having viral infection, having high body temperature, having a running nose, having a headache, fatigue, etc. Therefore, it might be that some plants used for fever treatment were not used as anti-viral purposes. We had information on Karen ethnomedicinal plants from 25 villages reported in 16 references including one book, one journal article, and 14 theses (Additional Data 1 AD1).

To rank the plants according to how important they were to the Karen for treating infectious diseases we calculated the Cultural Importance Index (CI) [47] as:

CI = UR/N

where UR is the total number of use reports for a species and N is the total number of informants in the interview. Some studies might use other indices such as Use Value (UV) [48], which is equivalent to CI, but they are calculated differently [47]. Because this study used ethnomedicinal metadata data, we used “Pseudoinformants” [15], which refer to a studied village or a data source, instead of a person who is giving ethnomedicinal data. A CI value of one would indicate that a species was mentioned for the same use in all 25 villages, whereas a use value close to zero would indicate that a species was mentioned for a particular use in only one or a few of the 25 villages. The uses, which were reported as symptoms of infectious diseases, were specified as they appeared in the original reports and then they were classified into infection types such as bacterial, fungal, viral, helminth worm, protozoal, and infestations from small animals (insects, ticks, lice, etc.), and unidentified (some symptoms caused by many infection types).

## 5. Conclusions

We identified medicinal plants used by the Karen for treatments of infectious diseases from 25 Karen villages in northern and western Thailand. We found 127 species in 59 families, which so far is the largest list of anti-infectious plants assembled for the Karen ethnic group. Among the families, Leguminosae, Euphorbiaceae, Asteraceae, Lauraceae, Apocynaceae, Menispermaceae, and Lamiaceae were the most commonly used. Among the species, *Cleidion javanicum*, *Tinospora crispa*, *Litsea cubeba*, *Aesculus assamica*, *Tadehagi triquetrum*, *Senna alata, Tithonia diversifolia*, *Embelia sessiliflora*, and *Combretum indicum* were the most commonly used. We recommend that these could be candidates for pharmacological research in discoveries of modern medicines for treatments of infectious diseases.

## Figures and Tables

**Table 1:** CI values for medicinal plant species used for treatment infection diseases by the Karen in Thailand.

Species	Bacterial infection	Fungal infection	Helminth worm infection	Protozoal infection	Small animal infection	Unidentified infection	Viral infection	Total CI value
*Acacia caesia* (L.) Willd.	0.04	-	0.04	-	-	-	0.04	0.12
*Acacia concinna* (Willd.) DC.	0.04	0.04	-	-	-	0.04	-	0.12
*Acalypha spiciflora* Burm.f.	-	-	-	0.04	-	-	-	0.04
*Achyranthes aspera* L.	-	-	-	-	-	-	0.04	0.04
*Acmella oleracea* (L.) R.K.Jansen	-	-	0.04	-	-	-	-	0.04
*Acmella paniculata* (Wall. ex DC.) R.K.Jansen	-	-	0.04	-	-	-	-	0.04
*Acorus calamus* L.	0.04	-	-	-	-	-	-	0.04
*Aesculus assamica* Griff.	0.08	-	-	-	-	-	0.04	0.12
*Ageratina adenophora* (Spreng.) R.M.King & H.Rob.	-	-	-	-	-	0.04	-	0.04
*Aloe vera* (L.) Burm.f.	-	-	-	-	-	-	0.04	0.04
*Alstonia rostrata* C. E. C. Fisch.	-	-	-	0.04	-	-	-	0.04
*Alstonia scholaris* (L.) R. Br.	-	-	-	0.08	-	-	-	0.08
*Amphineurion marginatum* (Roxb.) D.J.Middleton	-	-	0.04	-	-	-	-	0.04
*Angiopteris evecta* (G. Forst.) Hoffm.	0.04	-	-	-	-	-	-	0.04
*Annona squamosa* L.	-	-	-	-	0.04	-	-	0.04
*Archidendron jiringa* (Jack) I.C.Nielsen	-	-	-	-	-	0.08	-	0.08
*Arisaema auriculatum* Buchet	0.04	-	-	-	-	-	-	0.04
*Aristolochia tagala* Cham.	-	-	0.04	-	-	0.04	0.04	0.12
*Artemisia atrovirens* Hand.-Mazz.	-	-	-	-	-	-	0.04	0.04
*Artocarpus heterophyllus* Lam.	-	-	0.04	-	-	-	-	0.04
*Betula alnoides* Buch.-Ham. ex D.Don	-	-	-	-	-	-	0.04	0.04
*Blumea balsamifera* (L.) DC.	-	-	-	0.04	-	-	-	0.04
*Brucea javanica* (L.) Merr.	-	-	-	0.04	-	-	-	0.04
*Buddleja asiatica* Lour.	-	-	-	-	-	0.04	-	0.04
*Caesalpinia sappan* L.	0.04	-	-	-	-	-	-	0.04
*Cajanus cajan* (L.) Millsp.	0.04	-	-	-	0.04	0.04	-	0.12
*Cassytha filiformis* L.	-	-	-	-	-	-	0.04	0.04
*Celtis tetrandra* Roxb.	-	-	-	-	-	-	0.04	0.04
*Centella asiatica* (L.) Urb.	-	-	-	-	-	-	0.04	0.04
*Cheilocostus speciosus* (J.Koenig) C.D.Specht	-	-	-	-	-	0.04	-	0.04
*Chromolaena odorata* (L.) R.M.King & H.Rob.	-	0.04	-	0.04	-	-	-	0.08
*Chrozophora tinctoria* (L.) A.Juss.	-	-	0.04	-	-	-	-	0.04
*Cissampelos hispida* Forman	-	-	0.04	-	-	-	-	0.04
*Cissus javana* DC.	0.04	-	-	-	-	-	-	0.04
*Clausena excavata* Burm.f.	-	-	-	-	0.04	0.04	-	0.08
*Cleidion javanicum* Blume	0.08	-	-	0.16	-	-	0.08	0.32
*Codiaeum variegatum* (L.) Rumph. ex A.Juss.	0.04	-	-	-	-	-	-	0.04
*Combretum indicum* (L.) DeFilipps	-	-	0.08	-	-	-	-	0.08
*Croton sepalinus* Airy Shaw	-	-	-	0.04	-	-	-	0.04
*Cyclea barbata* Miers	0.04	-	-	-	-	-	-	0.04
*Cyclocodon lancifolius* subsp. *Celebicus* (Blume) K.E.Morris & Lammers	-	-	-	-	-	-	0.04	0.04
*Dactylicapnos scandens* (D.Don) Hutch.	-	-	-	-	-	0.04	-	0.04
*Dendrocalamus hamiltonii* Nees & Arn. ex Munro	-	0.04	-	-	-	-	-	0.04
*Derris elliptica* (Wall.) Benth.	-	-	-	-	0.04	-	-	0.04
*Dianella ensifolia* (L.) DC.	-	-	-	0.04	-	-	-	0.04
*Diospyros mollis* Griff.	-	-	0.04	-	-	-	-	0.04
*Drymaria cordata* (L.) Willd. ex Schult.	-	-	-	-	-	0.04	-	0.04
*Eichhornia crassipes* (Mart.) Solms	0.04	-	-	-	-	-	-	0.04
*Elephantopus scaber* L.	-	-	-	0.04	-	-	-	0.04
*Eleusine indica* (L.) Gaertn.	-	-	-	0.04	-	-	-	0.04
*Embelia sessiliflora* Kurz	-	-	0.16	-	-	-	-	0.16
*Entada rheedii* Spreng.	0.04	-	-	-	-	-	-	0.04
*Erythrina subumbrans* (Hassk.) Merr.	0.04	-	-	-	-	-	-	0.04
*Euphorbia heterophylla* L.	-	-	0.04	-	-	-	-	0.04
*Ficus fistulosa* Reinw. ex Blume	0.04	-	-	-	-	-	-	0.04
*Flemingia lineata* (L.) Aiton	0.04	-	-	-	-	-	-	0.04
*Flueggea leucopyrus* Willd.	-	-	-	-	-	-	0.08	0.08
*Glinus herniarioides* (Gagnep.) Tardieu	-	-	-	-	-	-	0.04	0.04
*Glochidion sphaerogynum* (MÃ_ll.Arg.) Kurz	-	-	0.04	-	-	-	-	0.04
*Gmelina arborea* Roxb.	-	0.04	-	-	-	-	-	0.04
*Grewia nervosa* (Lour.) Panigrahi	-	-	0.04	-	-	-	-	0.04
*Harrisonia perforata* (Blanco) Merr.	-	0.04	-	-	-	-	-	0.04
*Hedyotis ampliflora* Hance	-	0.04	-	-	-	-	-	0.04
*Helicteres elongata* Wall. ex Bojer	-	-	-	0.04	-	-	-	0.04
*Heliotropium indicum* L.	-	-	-	-	-	-	0.04	0.04
*Hydnocarpus ilicifolia* King	-	-	0.04	-	-	-	-	0.04
*Ixora henryi* H.Lév.	-	-	-	0.04	-	-	-	0.04
*Justicia gendarussa* Burm.f.	-	-	-	-	-	-	0.04	0.04
*Lepisanthes senegalensis* (Poir.) Leenh.	-	-	-	0.04	-	-	-	0.04
*Leucaena leucocephala* (Lam.) de Wit	-	-	0.04	-	-	-	-	0.04
*Litsea cubeba* (Lour.) Pers.	0.04	0.04	-	0.08	-	-	0.12	0.28
*Litsea glutinosa* (Lour.) C.B.Rob.	-	-	-	-	0.04	-	-	0.04
*Luffa cylindrica* (L.) M.Roem.	-	-	-	-	0.04	-	-	0.04
*Mallotus philippensis* (Lam.) Müll.Arg.	-	-	0.04	-	-	-	-	0.04
*Mangifera indica* L.	0.04	-	-	-	-	-	-	0.04
*Melia azedarach* L.	-	-	0.04	-	-	-	-	0.04
*Melicope glomerata* (W. G. Craib) T.G. Hartley	-	-	-	0.08	-	-	-	0.08
*Microcos paniculata* L.	-	-	0.04	-	-	-	-	0.04
*Millingtonia hortensis* L.f.	-	-	-	0.04	-	-	-	0.04
*Momordica charantia* L.	-	-	-	-	-	-	0.04	0.04
*Morus macroura* Miq.	0.04	-	-	-	-	-	-	0.04
*Mucuna macrocarpa* Wall.	0.04	-	-	-	-	-	-	0.04
*Mucuna pruriens* (L.) DC.	-	0.04	-	-	-	-	-	0.04
*Mussaenda sanderiana* Ridl.	-	-	-	-	-	-	0.04	0.04
*Ocotea lancifolia* (Schott) Mez	-	-	-	-	-	-	0.04	0.04
*Oroxylum indicum* (L.) Kurz	0.04	-	-	-	-	-	0.04	0.08
*Oxalis acetosella* L.	0.04	-	-	-	-	-	-	0.04
*Passiflora foetida* L.	-	-	0.04	-	-	-	-	0.04
*Peliosanthes macrophylla* Wall. ex Baker	-	-	0.04	-	-	-	-	0.04
*Peperomia pellucida* (L.) Kunth	-	-	0.04	-	-	-	-	0.04
*Persicaria barbata* (L.) H.Hara	-	-	-	0.04	-	-	-	0.04
*Phlogacanthus curviflorus* (Wall.) Nees	-	-	-	-	0.04	-	-	0.04
*Phymatopteris cruciformis* (Ching) Pic. Serm.	-	-	-	-	0.04	-	-	0.04
*Picrasma javanica* Blume	-	-	-	0.04	-	-	-	0.04
*Plectranthus scutellarioides* (L.) R.Br.	-	-	-	-	0.04	-	-	0.04
*Plumbago indica* L.	-	0.04	-	-	-	-	-	0.04
*Pothos chinensis* (Raf.) Merr.	0.04	-	-	-	-	-	-	0.04
*Psidium guajava* L.	-	-	-	0.04	-	-	-	0.04
*Pteridium aquilinum* Kuhn var. wightianum Tryon	0.04	-	-	-	-	-	-	0.04
*Rauvolfia serpentina* (L.) Benth. ex Kurz	-	-	-	0.08	-	-	-	0.08
*Rauvolfia verticillata* (Lour.) Baill.	-	-	-	0.12	-	-	-	0.12
*Rhinacanthus nasutus* (L.) Kurz	-	-	-	-	-	-	0.04	0.04
*Rotheca serrata* (L.) Steane & Mabb.	-	0.04	0.04	0.04	-	0.04	-	0.16
*Rubia cordifolia* L.	-	-	-	0.04	-	-	-	0.04
*Saccharum officinarum* L.	-	-	0.04	-	-	-	-	0.04
*Sambucus javanica* Blume	-	-	-	0.04	-	-	-	0.04
*Sapindus rarak* DC.	0.04	-	-	-	-	-	-	0.04
*Sarcandra glabra* (Thunb.) Nakai	-	-	0.04	-	-	-	-	0.04
*Scoparia dulcis* L.	0.04	0.04	-	0.04	-	-	-	0.12
*Senna alata* (L.) Roxb.	-	0.08	-	-	-	0.04	-	0.12
*Senna occidentalis* (L.) Link	-	-	-	0.04	-	-	-	0.04
*Smilax corbularia* Kunth	-	-	-	-	-	0.04	0.04	0.08
*Solanum indicum* L.	0.04	-	-	-	-	-	-	0.04
*Strobilanthes cusia* (Nees) Kuntze	0.04	-	-	-	-	-	-	0.04
*Styrax benzoides* W. G. Craib	-	-	-	0.04	-	-	-	0.04
*Syzygium fruticosum* DC.	-	-	-	-	-	0.04	-	0.04
*Tabernaemontana pandacaqui* Lam.	-	0.04	-	-	-	-	-	0.04
*Tadehagi triquetrum* (L.) H.Ohashi	0.08	-	0.08	-	-	-	-	0.16
*Tamarindus indica* L.	0.04	-	-	-	-	-	-	0.04
*Tectona grandis* L.f.	-	-	-	0.04	-	-	-	0.04
*Tinospora crispa* (L.) Hook. f. & Thomson	0.12	-	0.12	0.04	-	0.04	-	0.32
*Tithonia diversifolia* (Hemsl.) A.Gray	-	0.08	-	-	-	0.04	-	0.12
*Trevesia palmata* (Roxb. ex Lindl.) Vis.	-	-	0.04	-	-	-	-	0.04
*Triadica cochinchinensis* Lour.	-	-	-	0.04	-	-	0.04	0.08
*Trichosanthes tricuspidata* Lour.	-	-	-	0.04	-	-	-	0.04
Vitex trifolia L.	-	-	-	-	-	-	0.04	0.04
*Xylia xylocarpa* (Roxb.) Taub.	-	-	-	0.04	-	-	-	0.04

## Supplementary Materials

The following are available online at https://www.mdpi.com/2079-6382/9/6/298/s1, List of data sources: Table S1. CI values of medicinal plant families for treatment different infection types used by Karen in Thailand; Table S2. Karen ethnomedicinal plants for treatments of infectious diseases and fever.
