# Supplementary material for: Anti-Infectious Plants of the Thai Karen: A Meta-Analysis
Source: Antibiotics (Basel). 2020 Jun 2;9(6):298. doi: 10.3390/antibiotics9060298 (PMC7345792; doi:10.3390/antibiotics9060298)
Supplement: Supplementary file 1 [file antibiotics-09-00298-s001.pdf]

Supplementary Materials

Anti-infectious plants of the Thai Karen: A meta-analysis

Methee Phumthum and Henrik Balslev

List of data sources

1. Anderson EF. Plants and people of the Golden Triangle ethnobotany of the hill tribes of northern Thailand. Southwest Portland: Timber Press, Inc.,; 1993.
2. Junkhonkaen J. Ethnobotany of Ban Bowee, Amphoe Suan Phueng, Changwat Ratchaburi. Master thesis. Bangkok: Kasetsart University Library; 2012.
3. Junsongduang A. Roles and importance of sacred Forest in biodiversity conservation in Mae Chaem District, Chiang Mai Province. PhD thesis. Chiang Mai: Chiang Mai University. 2014.
4. Kaewsangsai S. Ethnobotany of Karen in Khun Tuen Noi Village, Mae Tuen Sub-district, Omkoi District, Chiang Mai Province. Master thesis. Chiang Mai: Chiang Mai University; 2017.
5. Kamwong K. Ethnobotany of Karens at Ban Mai Sawan and Ban Huay Pu Ling, Ban Luang Sub-District, Chom Thong District, Chiang Mai Province. Master thesis. Chiang Mai: Chiang Mai University; 2010.
6. Kantasrila, R. Ethnobotany fo Karen at Ban Wa Do Kro, Mae Song Sub-district, Tha Song Yang District, Tak Province. Master thesis. Chiang Mai: Chiang Mai University Library. 2016.
7. Klibai, A. Self-care with indigenous medicine of long-eared Karen ethnic group: Case study Ban Mae Sin, Ban Kang Pinjai, Ban Slok, Wang Chin district, Phrae province. Master thesis. Surin: Surin Rajabhat University. 2013.
8. Mahawongsanan, A., Change of herbal plants utilization of the Pgn K'nyau : A case study of Ban Huay Som Poy, Mae Tia Watershed, Chom Thong District, Chiang Mai Province. Master thesis. Chiang Mai: Chiang Mai University Library. 2008.
9. Prachuabaree L. Medicinal plants of Karang hill tribe in Baan Pong-lueg, Kaeng Krachan District, Phetchaburi Province. Master thesis, Pharmacy. Nakhonpathom: Silpakorn University; 2008.
10. Pongamornkul W. An ethnobotanical study of the Karen at Ban Yang Pu Toh and Ban Yang Thung Pong, Chiang Dao district, Chiang Mai province. Bachelor degree thesis. Chiang Mai: Chiang Mai University; 2003.
11. Puling W. Ethnobotany of Karen for studying medicinal plants at Angka Noi and Mae Klang villages, Chomthong district, Chiang Mai. Bachelor degree thesis. Chiang Mai: Chiang Mai University; 2001.
12. Sonsupub B. Ethnobotany of karen community in Raipa village, Huaykhayeng subdistrict, Thongphahpume district, Kanchanaburi province. Master thesis. Bangkok: Kasetsart University; 2010.
13. Sukkho T. A survey of medicinal plants used by Karen people at Ban Chan and Chaem Luang Subdidtricts, Mae Chaem district, Chiang Mai province. Master thesis. Chiang Mai: Chiang Mai University; 2008.
14. Sutjaritjai N, Wangpakapattanawong P, Balslev H, Inta A. Traditional Uses of Leguminosae among the Karen in Thailand. Plants. 2019;8(12).
15. Trisonthi S, Trisonthi P. Ethnobotany of Karen in Mae Hae Nua village, Mae Na Jorn subdistrict, Mae Chaem district, Chiang Mai. Report. Bangkok: CU Library; 1995.
16. Winijchaiyanan P. Ethnobotany of Karen in Chiang Mai. Master thesis. Chiang Mai: Chiang Mai University Library; 1995.

Anti-infectious plants of the Thai Karen: A meta-analysis

Methee Phumthum and Henrik Balslev

Table S1 CI values of medicinal plant families for treatment different infection types used by Karen in Thailand

Family	Bacterial infection	Fungal infection	Helminth worm infection	Protozoal infection	Small animal infection	Unidentified infection	Viral Infection
Acanthaceae	0.04	-	-	-	0.04	-	0.08
Achariaceae	-	-	0.04	-	-	-	-
Acoraceae	0.04	-	-	-	-	-	-
Adoxaceae	-	-	-	0.04	-	-	-
Amaranthaceae	-	-	-	-	-	-	0.04
Anacardiaceae	0.04	-	-	-	-	-	-
Annonaceae	-	-	-	-	0.04	-	-
Apiaceae	-	-	-	-	-	-	0.04
Apocynaceae	-	0.04	0.04	0.32	-	-	-
Araceae	0.08	-	-	-	-	-	-
Araliaceae	-	-	0.04	-	-	-	-
Aristolochiaceae	-	-	0.04	-	-	0.04	0.04
Asparagaceae	-	-	0.04	-	-	-	-
Asteraceae	-	0.12	0.08	0.12	-	0.08	0.04
Betulaceae	-	-	-	-	-	-	0.04
Bignoniaceae	0.04	-	-	0.04	-	-	0.04
Boraginaceae	-	-	-	-	-	-	0.04
Campanulaceae	-	-	-	-	-	-	0.04
Cannabaceae	-	-	-	-	-	-	0.04
Caryophyllaceae	-	-	-	-	-	0.04	-
Chloranthaceae	-	-	0.04	-	-	-	-
Combretaceae	-	-	0.08	-	-	-	-
Costaceae	-	-	-	-	-	0.04	-
Cucurbitaceae	-	-	-	0.04	0.04	-	0.04
Dennstaedtiaceae	0.04	-	-	-	-	-	-
Ebenaceae	-	-	0.04	-	-	-	-
Euphorbiaceae	0.12	-	0.12	0.28	-	-	0.12
Lamiaceae	-	0.08	0.04	0.08	0.04	0.04	0.04
Lauraceae	0.04	0.04	-	0.08	0.04	-	0.20
Leguminosae	0.44	0.16	0.16	0.08	0.08	0.20	0.04
Malvaceae	-	-	0.08	0.04	-	-	-
Marattiaceae	0.04	-	-	-	-	-	-
Meliaceae	-	-	0.04	-	-	-	-
Menispermaceae	0.16	-	0.16	0.04	-	0.04	-
Molluginaceae	-	-	-	-	-	-	0.04
Moraceae	0.08	-	0.04	-	-	-	-
Myrtaceae	-	-	-	0.04	-	0.04	-
Oxalidaceae	0.04	-	-	-	-	-	-

Papaveraceae	-	-	-	-	-	0.04	-
Passifloraceae	-	-	0.04	-	-	-	-
Phyllanthaceae	-	-	0.04	-	-	-	0.08
Piperaceae	-	-	0.04	-	-	-	-
Plantaginaceae	0.04	0.04	-	0.04	-	-	-
Plumbaginaceae	-	0.04	-	-	-	-	-
Poaceae	-	0.04	0.04	0.04	-	-	-
Polygonaceae	-	-	-	0.04	-	-	-
Polypodiaceae	-	-	-	-	0.04	-	-
Pontederiaceae	0.04	-	-	-	-	-	-
Primulaceae	-	-	0.16	-	-	-	-
Rubiaceae	-	0.04	-	0.08	-	-	0.04
Rutaceae	-	0.04	-	0.08	0.04	0.04	-
Sapindaceae	0.12	-	-	0.04	-	-	0.04
Scrophulariaceae	-	-	-	-	-	0.04	-
Simaroubaceae	-	-	-	0.08	-	-	-
Smilacaceae	-	-	-	-	-	0.04	0.04
Solanaceae	0.04	-	-	-	-	-	-
Styracaceae	-	-	-	0.04	-	-	-
Vitaceae	0.04	-	-	-	-	-	-
Xanthorrhoeaceae	-	-	-	0.04	-	-	0.04

Table S2 Karen ethnomedicinal plants for treatments of infectious diseases and fever

Species	Used for infections	Used for fever*	Used for both
Acacia caesia (L.) Willd.	✓		
Acacia concinna (Willd.) DC.	✓		
Acacia rugata (Lam.) Fawc. & Rendle		✓	
Acalypha spiciflora Burm.f.			✓
Achyranthes aspera L.	✓		
Acmella oleracea (L.) R.K.Jansen	✓		
Acmella paniculata (Wall. ex DC.) R.K.Jansen	✓		
Acorus calamus L.			✓
Aesculus assamica Griff.	✓		
Ageratina adenophora (Spreng.) R.M.King & H.Rob.	✓		
Aglaia lawii (Wight) C.J.Saldanha		✓	
Allium ascalonicum L.		✓	
Aloe vera (L.) Burm.f.			✓
Alstonia rostrata C. E. C. Fisch.			✓
Alstonia scholaris (L.) R. Br.			✓
Amphineurion marginatum (Roxb.) D.J.Middleton	✓		
Andrographis paniculata (Burm.f.) Nees		✓	
Angiopteris evecta (G. Forst.) Hoffm.	✓		
Annona squamosa L.	✓		
Aquilaria crassna Pierre ex Lecomte		✓	
Archidendron clypearia (Jack) I.C.Nielsen		✓	
Archidendron jiringa (Jack) I.C.Nielsen	✓		
Arisaema auriculatum Buchet	✓		
Aristolochia tagala Cham.	✓		
Artemisia atrovirens Hand.-Mazz.			✓
Artocarpus heterophyllus Lam.	✓		

Aspidistra elatior Blume		✓	
Azadirachta indica A.Juss.		✓	
Betula alnoides Buch.-Ham. ex D.Don	✓		
Blumea balsamifera (L.) DC.			✓
Brucea javanica (L.) Merr.	✓		
Buddleja asiatica Lour.	✓		
Caesalpinia sappan L.			✓
Cajanus cajan (L.) Millsp.	✓		
Calophyllum polyanthum Wall. ex Planch. & Triana		✓	
Camellia sinensis (L.) Kuntze		✓	
Cassytha filiformis L.			✓
Cayratia pedata (Lam.) Gagnep.		✓	
Celtis tetrandra Roxb.			✓
Celtis timorensis Span.		✓	
Centella asiatica (L.) Urb.			✓
Cheilocostus speciosus (J.Koenig) C.D.Specht	✓		
Chloranthus elatior Link		✓	
Chromolaena odorata (L.) R.M.King & H.Rob.			✓
Chrozophora tinctoria (L.) A.Juss.	✓		
Cinnamomum subavenium Miq.		✓	
Cissampelos hispida Forman			✓
Cissus javana DC.	✓		
Citrus aurantiifolia (Christm.) Swingle		✓	
Citrus hystrix DC.		✓	
Citrus maxima (Burm.) Merr.		✓	
Clausena excavata Burm.f.			✓
Clausena lenis Drake		✓	
Cleidion javanicum Blume			✓
Clematis smilacifolia Wall.		✓	
Clerodendrum infortunatum L.		✓	
Clerodendrum nutans Wall. ex Jack		✓	
Clerodendrum paniculatum L.		✓	
Clinacanthus nutans (Burm.f.) Lindau		✓	
Codiaeum variegatum (L.) Rumph. ex A.Juss.	✓		
Combretum indicum (L.) DeFilipps	✓		
Croton sepalinus Airy Shaw	✓		
Cyanthillium cinereum (L.) H.Rob.		✓	
Cyclea barbata Miers	✓		
Cyclocodon lancifolius subsp. *Celebicus* (Blume) K.E.Morris & Lammers	✓		
Dactylicapnos scandens (D.Don) Hutch.	✓		
Dalbergia cana Kurz		✓	
Dendrocalamus hamiltonii Nees & Arn. ex Munro	✓		
Dendrophthoe pentandra (L.) Miq.		✓	
Derris elliptica (Wall.) Benth.	✓		
Desmos dumosus (Roxb.) Saff.		✓	
Desmos macrocarpus Bân		✓	
Dianella ensifolia (L.) DC.	✓		
Diospyros mollis Griff.	✓		
Dracaena terniflora Roxb.		✓	
Drymaria cordata (L.) Willd. ex Schult.	✓		

Dysoxylum grande Hiern		✓	
Eichhornia crassipes (Mart.) Solms	✓		
Elephantopus scaber L.			✓
Eleusine indica (L.) Gaertn.	✓		
Eleutherine bulbosa (Mill.) Urb.		✓	
Elytranthe albida (Blume) Blume		✓	
Embelia sessiliflora Kurz	✓		
Entada rheedii Spreng.	✓		
Erythrina subumbrans (Hassk.) Merr.	✓		
Eupatorium fortunei Turcz.		✓	
Euphorbia heterophylla L.	✓		
Eurycoma longifolia Jack		✓	
Falconeria insignis Royle		✓	
Ficus fistulosa Reinw. ex Blume	✓		
Flacourtia indica (Burm.f.) Merr.		✓	
Flemingia lineata (L.) Aiton	✓		
Flueggea leucopyrus Willd.	✓		
Garuga pinnata Roxb.		✓	
Glinus herniarioides (Gagnep.) Tardieu	✓		
Glochidion sphaerogynum (MÃ_ll.Arg.) Kurz	✓		
Gmelina arborea Roxb.	✓		
Grewia nervosa (Lour.) Panigrahi	✓		
Harrisonia perforata (Blanco) Merr.			✓
Hedyotis ampliflora Hance			✓
Hedyotis pruinosa Wight & Arn.		✓	
Helicteres elongata Wall. ex Bojer	✓		
Heliotropium indicum L.	✓		
Holarrhena pubescens Wall. ex G.Don		✓	
Houttuynia cordata Thunb.		✓	
Hydnocarpus ilicifolia King	✓		
Illigera trifoliata (Griff.) Dunn		✓	
Imperata cylindrica (L.) Raeusch.		✓	
Indigofera tinctoria L.		✓	
Inula cappa (Buch.-Ham. ex D.Don) DC.		✓	
Ixora henryi H.Lév.			✓
Jasminum laurifolium Roxb. ex Hornem.		✓	
Justicia gendarussa Burm.f.			✓
Lepisanthes senegalensis (Poir.) Leenh.	✓		
Leucaena leucocephala (Lam.) de Wit	✓		
Litsea cubeba (Lour.) Pers.	✓		
Litsea glutinosa (Lour.) C.B.Rob.	✓		
Litsea monopetala (Roxb.) Pers.		✓	
Luffa cylindrica (L.) M.Roem.	✓		
Mallotus philippensis (Lam.) Müll.Arg.			✓
Mangifera indica L.	✓		
Mayodendron igneum (Kurz) Kurz		✓	
Melastoma sanguineum Sims.		✓	
Melia azedarach L.	✓		
Melicope glomerata (W. G. Craib) T.G. Hartley			✓
Memecylon pauciflorum Blume		✓	
Microcos paniculata L.	✓		

Micromelum integerrimum (Buch.-Ham. ex DC.) Wight & Arn. ex M. Roem.	✓		
Millingtonia hortensis L.f.			✓
Mimosa diplotricha Sauvalle		✓	
Mimosa pigra L.		✓	
Mimosa pudica L.		✓	
Molineria capitulata (Lour.) Herb.		✓	
Momordica charantia L.	✓		
Morus macroura Miq.	✓		
Mucuna macrocarpa Wall.	✓		
Mucuna pruriens (L.) DC.	✓		
Musa acuminata Colla		✓	
Mussaenda sanderiana Ridl.	✓		
Nyctocalos brunfelsiiflora Teijsm. & Binn.		✓	
Ocotea lancifolia (Schott) Mez	✓		
Oenanthe javanica (Blume) DC.		✓	
Oroxylum indicum (L.) Kurz	✓		
Osbeckia chinensis L.		✓	
Oxalis acetosella L.	✓		
Passiflora foetida L.	✓		
Peliosanthes caesia J.M.H.Shaw		✓	
Peliosanthes macrophylla Wall. ex Baker			✓
Peperomia pellucida (L.) Kunth	✓		
Persicaria barbata (L.) H.Hara	✓		
Phlogacanthus curviflorus (Wall.) Nees			✓
Phyllanthus amarus Schumach. & Thonn.		✓	
Phymatopteris cruciformis (Ching) Pic. Serm.	✓		
Physalis minima L.		✓	
Picrasma javanica Blume			✓
Plantago major L.		✓	
Platycerium wallichii Hook.		✓	
Plectranthus amboinicus (Lour.) Spreng.		✓	
Plectranthus scutellarioides (L.) R.Br.	✓		
Plumbago indica L.			✓
Plumeria obtusa L.		✓	
Polygala arillata Buch.-Ham. ex D. Don		✓	
Pothos chinensis (Raf.) Merr.			✓
Pothos scandens L.		✓	
Psidium guajava L.			✓
Pteridium aquilinum Kuhn var. wightianum Tryon	✓		
*Pteridrys syr*matica (Willd.) C. Chr. & Ching		✓	
Rauvolfia serpentina (L.) Benth. ex Kurz	✓		
Rauvolfia verticillata (Lour.) Baill.	✓		
Rhinacanthus nasutus (L.) Kurz	✓		
Rotheca serrata (L.) Steane & Mabb.			✓
Rubia cordifolia L.	✓		
Saccharum officinarum L.	✓		
Sambucus javanica Blume			✓
Sapindus rarak DC.	✓		
Sarcandra glabra (Thunb.) Nakai			✓
Scadoxus multiflorus (Martyn) Raf.		✓	

Schima wallichii Choisy		✓	
Scoparia dulcis L.			✓
Scurrula ferruginea (Jack) Danser		✓	
Senna alata (L.) Roxb.	✓		
Senna hirsuta (L.) H.S.Irwin & Barneby		✓	
Senna occidentalis (L.) Link	✓		
Sida acuta Burm.f.		✓	
Sida cordifolia L.		✓	
Sida rhombifolia L.		✓	
Smilax corbularia Kunth	✓		
Smilax glabra Roxb.		✓	
Smilax luzonensis C.Presl		✓	
Solanum indicum L.	✓		
Spondias pinnata (L. f.) Kurz		✓	
Streblus asper Lour.		✓	
Strobilanthes cusia (Nees) Kuntze			✓
Styrax benzoides W. G. Craib	✓		
Syzygium fruticosum DC.	✓		
Tabernaemontana pandacaqui Lam.	✓		
Tadehagi triquetrum (L.) H.Ohashi			✓
Tamarindus indica L.			✓
Tectona grandis L.f.	✓		
Thunbergia coccinea Wall.		✓	
Thunbergia laurifolia Lindl.		✓	
Thysanolaena latifolia (Roxb. ex Hornem.) Honda		✓	
Tiliacora triandra Diels		✓	
Tinospora baenzigeri Forman		✓	
Tinospora crispa (L.) Hook. f. & Thomson			✓
Tithonia diversifolia (Hemsl.) A.Gray	✓		
Trevesia palmata (Roxb. ex Lindl.) Vis.	✓		
Triadica cochinchinensis Lour.			✓
Trichosanthes tricuspidata Lour.	✓		
*Turpinia pom*ifera (Roxb.) DC.		✓	
Viscum articulatum Burm. f.		✓	
Vitex trifolia L.			✓
Xylia xylocarpa (Roxb.) Taub.	✓		

*Data from Phumthum and Sadgrove (2020)
